# LINC00461 facilitates HNSCC development and reduces chemosensitivity by impairing miR-195-mediated inhibition of HOXA10

**DOI:** 10.1016/j.omto.2021.01.008

**Published:** 2021-01-20

**Authors:** Yifang Guan, Aizhong Guan, Long Chen, Aimei Gong

**Affiliations:** 1 Department of Stomatology, Linyi People’s Hospital, Linyi 276000, Shandong, P.R. China

**Keywords:** head and neck squamous cell carcinoma, long intergenic noncoding RNA 00461, microRNA-195, homeobox A10, epithelial-mesenchymal transition, chemoresistance

## Abstract

Homeobox A10 (HOXA10) has been regarded to serve as an oncogene in head and neck squamous cell carcinoma (HNSCC). This study was intended to explore the interaction among the long intergenic noncoding RNA 00461 (LINC00461), microRNA (miR)-195, and HOXA10, and to investigate its role in epithelial-mesenchymal transition (EMT) and chemoresistance in HNSCC. The effects of LINC00461, miR-195, and HOXA10 on the EMT and chemoresistance of HNSCC cells were analyzed by comprehensive analysis of gain- and loss-of-function techniques. The intimate relationships among LINC00461, miR-195, and HOXA10 were investigated by several procedures such as RNA-binding protein immunoprecipitation, RNA pull-down, and dual-luciferase reporter assays. A xenotransplantation tumor model in nude mice was established for the assessment of the tumorigenic ability of the cells *in vivo*. Our findings indicated that LINC00461 was highly expressed in HNSCC and its overexpression induced EMT and precipitated the chemoresistance of HNSCC cells to cisplatin. The LINC00461 could bind to miR-195 while miR-195 targeted HOXA10 independently. Moreover, LINC00461 impaired miR-195-mediated inhibition of HOXA10 to induce EMT and increase the chemoresistance in HNSCC. Tumor weight and volume were reduced by lentivirus-mediated elevation of miR-195 by inhibition of HOXA10, which could be annulled by LINC00461 overexpression. LINC00461 downregulates the expression of miR-195 to subsequently upregulate the expression of HOXA10, thereby promoting EMT and enhancing chemoresistance in HNSCC.

## Graphical Abstract

**Figure:**
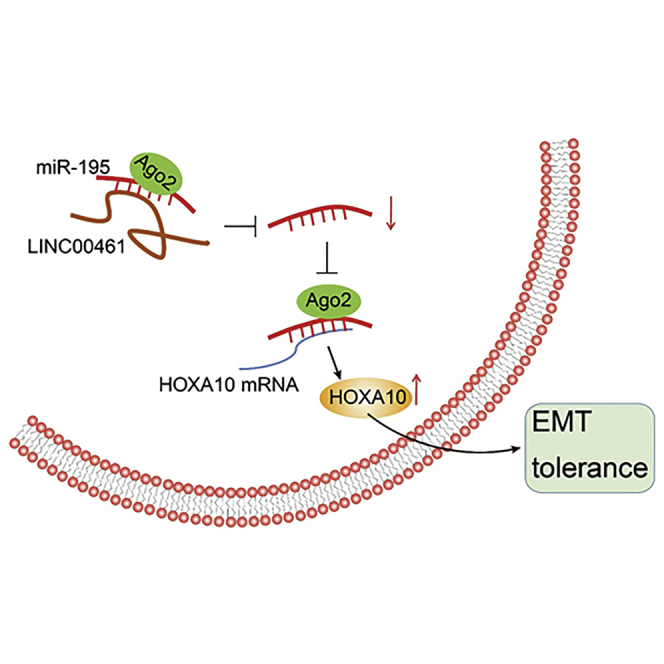

## Teaser

Chemotherapy and radiation are the current common methods to eliminate the remaining cancer cells of head and neck squamous cell carcinoma (HNSCC), but resistance ultimately leads to the failure of treatment. We found that LINC00461 could enhance chemoresistance in HNSCC, revealing a selective vulnerability of HNSCC.

## Introduction

Head and neck squamous cell carcinoma (HNSCC) is a heterogeneous group of tumors that ranks as the 6^th^ most prevalent malignancy worldwide.^1^ HNSCC manifests in the squamous epithelium of the head and neck regions and is classified into the following types: tongue SCC (TSCC), oral SCC (OSCC), laryngeal SCC (LSCC), and nasopharyngeal carcinoma (NPC).^2^ The vital associated risk factors for HNSCC include exposure to environmental carcinogens such as tobacco and alcohol consumption and contracting the Epstein-Barr virus (HBV) and human papillomaviruses.^3^ Moreover, HNSCC, with its alarmingly high incidence, affects >800,000 people annually worldwide.^4^ The currently adopted standard clinical protocol for HNSCC is surgical intervention in combination with chemotherapy and radiation to eliminate any residual cancer cells.^5^ However, the use of combination therapy is a promising aspect of oncology due to the underlying complexity of the immune system and various therapies for tumor evasion.^6^ Despite advancements in treatment, the development of resistance to cisplatin by tumor cells accounts for the failure of treatment protocols.^5^^,^^7^

Notably, long non-coding RNAs (lncRNAs) have been identified to be dysregulated in HNSCC.^8^ lncRNAs, non-coding RNAs longer than 200 nt, can serve as potential biomarkers in the diagnosis, prognosis, and targeted therapy of different types of cancers.^9^ LINC00461 has been identified in the intergenic region of human chromosome 5 and reported to function as a vital regulator of cancer; for instance, it has been identified to function as a promoter in the progression of glioma.^10^ In addition, LINC00461 possesses an oncogenic role in colorectal cancer cells by targeting microRNA (miR)-323b-3p in the nuclear factor I B (NFIB) signaling pathway.^11^ Nevertheless, its role in HNSCC remains undefined.

lncRNAs can serve as regulators of microRNAs (miRNAs), thereby altering the expression of their target mRNAs.^12^ miRNAs are small, non-coding molecules that regulate the gene expression at the transcription and post-transcriptional levels.^13^ miR-195, a member of the miR-15/107 family, is regarded as an important therapeutic target or biomarker for head and neck cancer.^14^ For example, miR-195 inhibits the proliferative capability of and resistance to apoptosis of TSCC cells.^15^ The ability of miR-195 to serve as a tumor suppressor has been identified in LSCC.^16^

Thus, in the present study, we further determine the anti-tumor role of miR-195 in HNSCC. Accordingly, previously reported analysis from the bioinformatics website TargetScan, illustrated that homeobox A10 (HOXA10) is a target of miR-195. The HOX gene family has been regarded as trivial, with vital functionality in tumorigenesis.^17^^,^^18^ Principally, HOXA10 is implicated in gastric carcinogenesis due to its activation in gastric cancer.^19^ However, inhibition of HOXA10 induced by miR-135a-5p restrains the carcinogenesis in HNSCC.^20^ Consistently, our study demonstrated that LINC00461 could modulate the expression of HOXA10 by regulating miR-195, thus affecting the epithelial-mesenchymal transition (EMT) and chemoresistance of HNSCC cells.

## Results

### The potential functional significance of LINC00461/miR-195/HOXA10 in HNSCC

**Figure 1:**
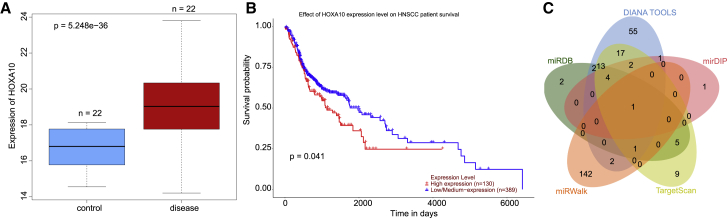
LINC00461 may regulate HOXA10 through binding to miR-195 in HNSCC (A) The boxplot of HOXA10 expression pattern in HNSCC from dataset GSE108061; the blue box at left refers to HOXA10 expression pattern in normal tissues and red box at right refers to HOXA10 expression pattern in the HNSCC tissues (p = 5.248e–36). The sample size for both control and HNSCC tumor is 22, and the y axis represents the expression pattern of HOXA10 relative to housekeeping genes. (B) The survival curves concerning the HOXA10 expression pattern obtained from the TCGA database (https://portal.gdc.cancer.gov), as analyzed by the ualcan tool (http://ualcan.path.uab.edu) (p = 0.041). High expression pattern indicates the TPM value greater than or equal to the upper quartile- low/moderate expression pattern indicates the TPM value less than the upper quartile. (C) Venn diagram of the miRNAs that could regulate HOXA10 predicted by the miRDB (http://www.mirdb.org) (dark green), DIANA TOOLS (http://diana.imis.athena-innovation.gr/DianaTools) (blue), mirDIP (http://ophid.utoronto.ca/mirDIP/) (pink), TargetScan (http://www.targetscan.org/vert_71/) (grass green), and miRWalk databases (http://mirwalk.umm.uni-heidelberg.de) (orange).

The differentially expressed genes (DEGs) in HNSCC were identified from the GSE108061 dataset retrieved from the Gene Expression Omnibus (GEO) database (https://www.ncbi.nlm.nih.gov/gds), which revealed that HOXA10 was significantly upregulated gene in HNSCC (Figure 1A). Using the online tool ualcan (http://ualcan.path.uab.edu), the clinical survival curves regarding the HOXA10 expression pattern in The Cancer Genome Atlas (TCGA) database (https://portal.gdc.cancer.gov) was analyzed, and our results revealed that a high expression pattern of HOXA10 was associated with the increased mortality of HNSCC (Figure 1B) (high expression indicates that the TPM value is greater than or equal to the upper quartile; low/moderate expression indicates that the TPM value is less than the upper quartile). According to Guo et al.^20^, HOXA10 can be regulated by miRNAs and aids the inhibition of the proliferation of HNSCC. In the present study, the potential upstream miRNA of HOXA10 was predicted by a combination of the miRDB (http://www.mirdb.org), DIANA TOOLS (http://diana.imis.athena-innovation.gr/DianaTools), mirDIP (http://ophid.utoronto.ca/mirDIP/), TargetScan (http://www.targetscan.org/vert_71/), and miRWalk databases (http://mirwalk.umm.uni-heidelberg.de), with a key intersected miRNA, namely miR-195, identified by the Venn diagram (Figure 1C). The binding site between miR-195 and HOXA10 was identified by the TargetScan database (Figure S1A).

Our results from the RNA22 database (https://cm.jefferson.edu/rna22/) predicted that LINC00461 could bind to miR-195 (Figure S1B). Accordingly, LINC00461 has been reported to mediate the miRNA expression pattern by competitively binding to miRNA, thus altering the overall survival of patients suffering from renal cell carcinoma.^21^ Hence, we speculated that LINC00461 may affect HNSCC progression via the regulation of HOXA10 expression pattern by binding to miR-195.

### LINC00461 and HOXA10 are highly expressed, while miR-195 is poorly expressed in HNSCC

**Figure 2:**
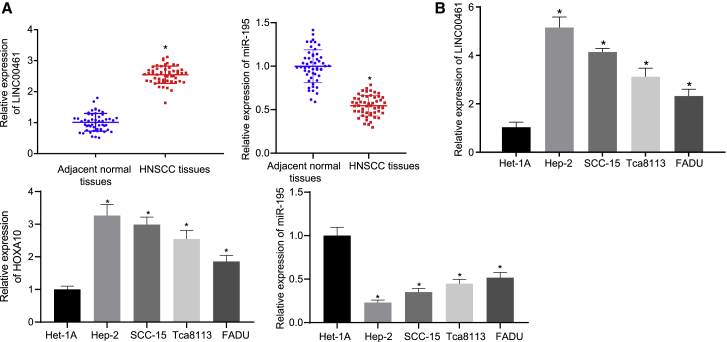
High expression pattern of LINC00461 and HOXA10, yet poor miR-195 expression in HNSCC (A) Expression pattern of LINC00176, HOXA10, and miR-195 in the adjacent normal (n = 52) and HNSCC tissues (n = 52). (B) Expression pattern of LINC00461, miR-195, and HOXA10 in the normal head and neck squamous cell lines and HNSCC cell lines. All of the data were measurement data expressed as means ± standard deviations. The data between HNSCC tissues and adjacent normal tissues were compared by paired t test. The data among multiple groups were compared by 1-way ANOVA. The experiment was conducted 3 times independently. ∗p < 0.05 versus adjacent normal tissues or Het-1A cells.

The expression patterns of LINC00461, HOXA10, and miR-195 in HNSCC and the adjacent normal tissues (collected from 52 patients with HNSCC) was determined by quantitative reverse transcription polymerase chain reaction (qRT-PCR), which showed that the expression patterns of LINC00461 and HOXA10 were higher, while that of miR-195 was lower in the HNSCC tissues compared to the adjacent normal tissues (Figure 2A; Table 1). qRT-PCR showed high expression patterns of LINC00461 and HOXA10, along with a diminished miR-195 expression pattern in the HNSCC cell lines (Hep-2, SCC-15, Tca8113, and FADU) as compared to the normal esophageal squamous cell line Het-1A (Figure 2B). Coherently, upregulated LINC00461 and HOXA10 and downregulated miR-195 were evident in the HNSCC cells.

**Table 1:** Individual expression values of LINC00461 and miR-195 in 52 HNSCC patients

Patients	Expression value (adjacent normal tissue/HNSCC tissue)	
	LINC00461	miR-195
1	1/2.353	1/0.404
2	0.55/2.589	1.224/0.786
3	0.767/2.635	1.119/0.571
4	0.74/2.709	0.686/0.58
5	0.914/2.585	1.304/0.544
6	1.075/2.822	0.904/0.483
7	0.88/2.262	0.836/0.624
8	1.453/2.692	1.415/0.467
9	0.67/3.024	1.047/0.72
10	0.656/2.855	1.079/0.517
11	1.138/2.383	0.949/0.521
12	0.751/2.775	1.157/0.546
13	0.9/2.322	1.101/0.596
14	1.301/2.141	0.749/0.61
15	1.261/2.847	1.093/0.526
16	1.052/2.303	0.718/0.354
17	0.917/2.35	0.764/0.474
18	1.132/2.806	0.96/0.689
19	1.259/2.61	1.091/0.483
20	1.148/2.493	0.754/0.481
21	0.856/2.563	0.59/0.588
22	0.728/2.497	1.003/0.719
23	1.1/2.92	0.965/0.631
24	0.936/2.336	0.909/0.702
25	1.055/2.74	0.967/0.329
26	1.042/2.665	0.866/0.38
27	1.798/2.337	1.036/0.336
28	1.049/2.504	1.365/0.508
29	0.792/3.119	1.14/0.542
30	0.912/2.668	1.282/0.416
31	0.512/2.607	1.277/0.411
32	0.735/2.879	1.196/0.744
33	0.704/2.88	1.057/0.297
34	0.683/2.63	1.245/0.583
35	1.346/2.151	0.982/0.54
36	1.323/2.323	1.282/0.577
37	1.106/2.673	0.988/0.664
38	0.938/2.329	0.616/0.653
39	0.933/1.637	0.986/0.64
40	1.089/2.553	0.961/0.438
41	1.392/3.065	0.99/0.4
42	0.702/2.412	0.976/0.659
43	1.085/2.596	0.889/0.682
44	1.397/2.117	0.918/0.71
45	0.79/2.719	0.939/0.574
46	1.279/2.591	1.026/0.641
47	0.537/2.301	1.036/0.652
48	1.681/2.436	0.795/0.584
49	1.185/2.441	1.033/0.461
50	1.118/2.517	0.83/0.46
51	0.892/2.037	0.717/0.431
52	1.427/2.596	1.133/0.464

The qRT-PCR expression value of each sample is related to U6/ glyceraldehyde-3-phosphate dehydrogenase (GAPDH).

### LINC00461 promotes EMT in HNSCC

**Figure 3:**
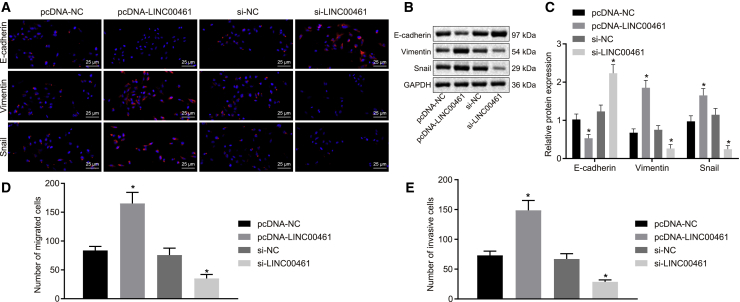
LINC00461 induces the EMT process in HNSCC (A) Immunofluorescence detection of EMT-related factors E-cadherin, Snail, and Vimentin in the HNSCC cell line FADU (400×). Three groups of red fluorescence represent E-cadherin, Snail, and Vimentin proteins, respectively, and blue fluorescence represents nuclei. (B) The protein bands of EMT-related factors E-cadherin, Snail, and Vimentin in the HNSCC cell line FADUs. (C) Quantitative analysis of (B). (D) The number of migrated FADU cells. (E) The number of invasive FADU cells. All of the data are measurement data and expressed as means ± standard deviations. The data among multiple groups were compared using 1-way ANOVA. The experiment was conducted 3 times independently. ∗p < 0.05 versus the pcDNA-NC group or the si-NC group.

EMT has been clinically adopted and evolved in human cancers, and the identification of the signaling pathways that lead to activation of the EMT protocols serve as promising therapeutic interventions.^22^, ^23^, ^24^ Meanwhile, E-cadherin, Vimentin, and Snail proteins serve as EMT markers for EMT induction.^23^^,^^25^ In the HNSCC cell line FADU, the expression pattern of several EMT markers (i.e., E-cadherin, Vimentin, and Snail) was detected by immunofluorescence and western blot analysis to evaluate the induction of EMT. As shown in Figures 3A–3C, the protein expression pattern of E-cadherin was reduced, whereas the expression patterns of Vimentin, Snail, and LINC00461 were significantly increased in cells transfected with pcDNA-LINC00461. Concurrently, the protein expression pattern of E-cadherin was elevated, whereas the expression patterns of Vimentin and Snail were reduced in cells transfected with small interfering RNA (siRNA) targeting LINC00461 (si-LINC00461). Transwell assay (Figures 3D and 3E) revealed that the cell migration and invasion abilities were enhanced by LINC00461 overexpression but reduced by the silencing of LINC00461. These aforementioned results indicated that LINC00461 promoted EMT in the HNSCC cells.

### Silencing of LINC00461 reduces chemoresistance of HNSCC cells to cisplatin

**Figure 4:**
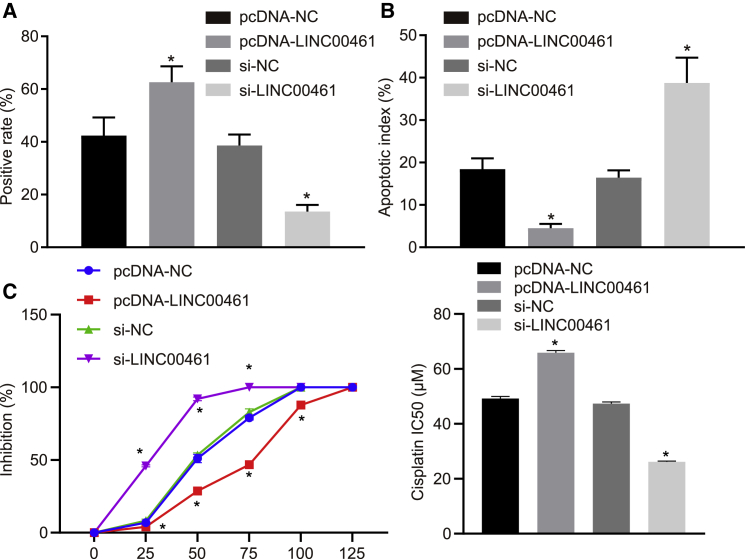
Silencing of LINC00461 inhibits chemoresistance of HNSCC to cisplatin (A) The proliferation of HNSCC cell line FADU/DDP. Red fluorescence represents proliferated cells and blue fluorescence represents nuclei. (B) The apoptosis rate of HNSCC cell line FADU/DDP. (C) The resistance to cisplatin in HNSCC cell line FADU/DDP. All of the data were measurement data expressed as means ± standard deviations. The data among multiple groups were compared using 1-way ANOVA. The experiment was conducted 3 times independently. ∗p < 0.05 versus the pcDNA-NC group or the si-NC group.

The EMT process is essentially involved in the process of the therapeutic resistance of cancer cells.^26^ As one of the gold standard methods for cell proliferation detection, 5-ethynyl-2′-deoxyuridine (EdU) assay can detect the highly sensitive proliferating cells and promote continuous cell-cycle assessment.^27^ Terminal deoxynucleotidyl transferase dUTP (2′-deoxyuridine,5′-triphosphate) nick end labeling (TUNEL) assay was based on the ability of terminal deoxynucleotidyl transferase (TdT) to label the blunt ends of double-stranded DNA fragments independent of a template.^28^ Thus, we also attempted to study the effect of LINC00461 on the chemoresistance of HNSCC cells in the cisplatin-resistant HNSCC cell line (FADU/DDP). The proliferation and apoptosis of FADU/DDP cells following the overexpression or silencing of LINC00461 were measured by EdU assay (Figure 4A) and TUNEL assay (Figure 4B), respectively. Our results depicted that the overexpression of LINC00461 led to a significant increase in cell proliferation and a significant decrease in the apoptosis rate. The proliferation ability was lowered, whereas the apoptosis rate was increased following transfection with si-LINC00461. Conjointly, the knockdown of LINC00461 reduced chemoresistance to 50 μM cisplatin in the HNSCC cells. After LINC00461 overexpression or silencing, drug sensitivity tests were performed to obtain the cisplatin half-maximal inhibitory concentration (IC_50_) values. The results illustrated the elevated resistance to cisplatin in HNSCC cells following LINC00461 overexpression, while contradictory results were found in cells following LINC00461 knockdown (Figure 4C). Thus, it may be plausible to suggest that silencing of LINC00461 could attenuate the restrictive chemoresistance of HNSCC cells to cisplatin.

### LINC00461 competitively binds to miR-195 and thus regulates HOXA10 expression

**Figure 5:**
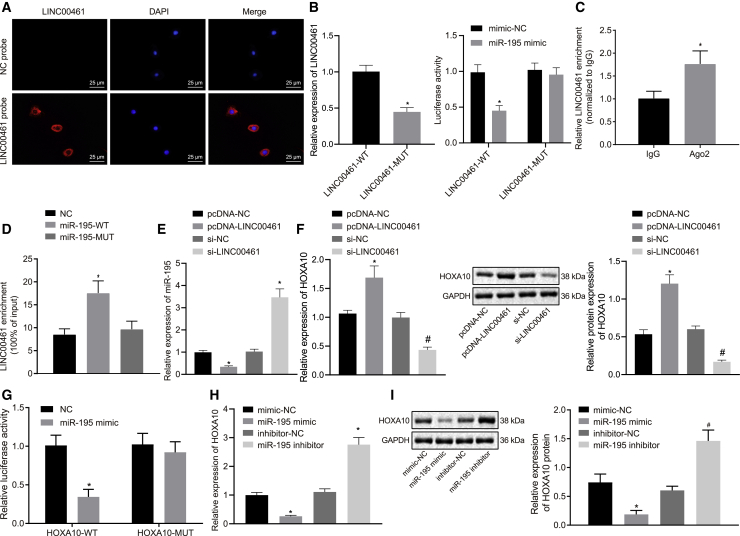
LINC00461 regulates the HOXA10 expression pattern by competitively binding to miR-195 (A) LINC00461 subcellular localization (400×). (B) The luciferase activity of LINC00461-WT and LINC00461-MUT in cells transfected with miR-195 mimic. (C) The binding of LINC00461 and Ago2 protein. (D) The binding of LINC00461 to miR-195. (E) The expression pattern of miR-195 in FADU cells overexpressing LINC00461. (F) The expression pattern of HOXA10 after overexpression or silencing LINC00461 in FADU cells. (G) The relationship between HOXA10 and miR-195. (H) The mRNA expression pattern of HOXA10 in FADU cells with miR-195 mimic. (I) Western blot analysis of the HOXA10 protein in FADU cells with miR-195 mimic. All of the data were measurement data expressed as means ± standard deviations. The data comparison between the 2 groups was performed with unpaired t test, and data among multiple groups were compared using 1-way ANOVA. The experiment was conducted 3 times independently. In (C), ∗p < 0.05 versus the IgG group. In (D) and (G), ∗p < 0.05 versus the NC group. In (E), ∗p < 0.05 versus the pcDNA-NC group; #p < 0.05 versus the si-NC group. In (B), (H), and (I), ∗p < 0.05 versus the mimic-NC group; #p < 0.05 versus the inhibitor-NC group.

The fluorescence *in situ* hybridization (FISH) assay was performed to localize the specific nucleic acid sequences in native context.^29^ We then studied the mechanism of LINC00461 in the context of HNSCC. The FISH assay validated no fluorescence of LINC00461 in the negative control (NC) probe, blue nuclei stained with 4′-6-diamidino-2-phenylindole (DAPI), and blue fluorescence in the nuclei in Merge. In contrast, in the LINC00461 probe, LINC00461 showed red fluorescence, and the blue nuclei stained with DAPI, while red fluorescence was primarily in the cytoplasm in Merge (Figure 5A). These findings suggested that LINC00461 was principally localized in the cytoplasm of the cells. Our results from the biological prediction website RNA22 identified the presence of a specific binding region between LINC00461 and the miR-195 sequences (Figure S1B), suggesting that LINC00461 could bind to miR-195. In addition, LINC00461 was predominantly expressed in the cytoplasm. Therefore, we speculated that LINC00461 may participate in the development of HNSCC by competitively binding to miR-195. The dual-luciferase reporter assay could serve as a tool to efficiently characterize the minimum promoter region.^30^ The dual-luciferase reporter assay was conducted to validate the binding of LINC00461 to miR-195, which showed that the luciferase activity of LINC00461-WT (wild type) was inhibited, while that of LINC00461-MUT (mutant) remained unaffected in cells transfected with miR-195 mimic (Figure 5B). Next, the interaction between LINC00461 and Argonaute2 (Ago2) and between LINC00461 and miR-195 was analyzed using RNA-binding protein immunoprecipitation (RIP) and RNA pull-down assays, respectively. RIP assay showed increased enrichment of LINC00461 binding to Ago2 (Figure 5C), indicating that LINC00461 can bind to the Ago2 protein. Moreover, the RNA pull-down assay revealed a significant increase in the LINC00461 level pulled down by miR-195- WT compared to that pull-down by NC and miR-195-MUT (Figure 5D). The expression pattern of miR-195 was reduced in cells overexpressing LINC00461, while it was increased upon LINC00461 silencing (Figure 5E). The aforementioned data supported the ability of LINC00461 to competitively bind to miR-195. When LINC00461 was overexpressed or silenced, the expression pattern of HOXA10 was determined by qRT-PCR and western blot analysis, and the results revealed a positive correlation between LINC00461 with the expression pattern of HOXA10 (Figure 5F). The silencing of HOXA1 upregulated the expression pattern of miR-195, while slightly inhibiting the expression pattern of the LINC00461. The microRNA.org database identified the presence of a specific miR-195 binding region in the 3′ UTR of the HOXA10 mRNA, thereby suggesting HOXA10 as a target gene of miR-195. Then the dual-luciferase reporter assay showed that the luciferase activity of the HOXA10-WT was inhibited by miR-195 mimic compared to the mimic NC, whereas HOXA10-MUT was not affected (Figure 5G). The mRNA and protein expression pattern of HOXA10 was measured by qRT-PCR and western blot analysis in cells following transfection with miR-195 mimic or inhibitor. As shown in Figures 5H and 5I, compared to the cells transfected with mimic-NC, the mRNA and protein expression pattern of HOXA10 was significantly decreased in cells transfected with miR-195 mimic. In comparison with the cells transfected with inhibitor-NC, the mRNA and protein expression patterns of HOXA10 were increased in cells transfected with the miR-195-inhibitor. Thus, miR-195 could specifically bind to HOXA10 3′ UTR and downregulate its expression at the post-transcriptional level. Collectively, LINC00461 could serve as a regulator of HOXA10 expression by competitively binding to miR-195.

### LINC00461 upregulates HOXA10, thus promoting EMT by inhibiting miR-195

**Figure 6:**
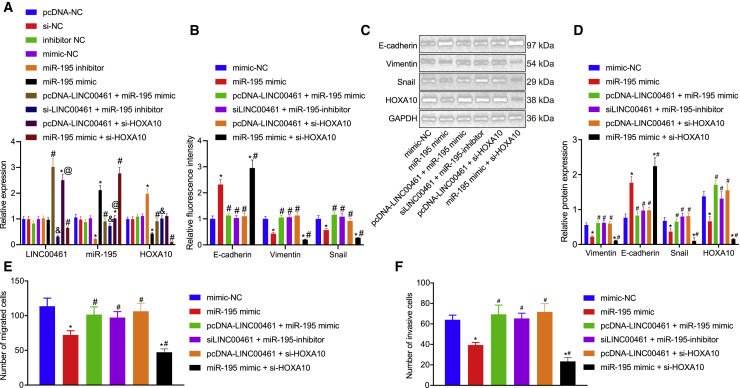
The LINC00461/miR-195/HOXA10 axis mediates the EMT process in HNSCC (A) The expression of LINC00461, miR-195 and HOXA10 in the FADU cells. (B) Statistical graph of the expression of EMT-related factors E-cadherin, N-cadherin, and Vimentin in the FADU cells. (C) Protein bands of EMT-related factors E-cadherin, N-cadherin, and Vimentin in the FADU cells. (D) Quantitative analysis of (C). (E) Transwell assay showing the migration ability of the FADU cells. (F) Transwell assay showing the invasion ability of the FADU cells. All of the data were measurement data expressed as means ± standard deviations. The data among multiple groups were compared using 1-way ANOVA. The experiment was conducted 3 times independently. ∗p < 0.05 versus the mimic-NC group. #p < 0.05 versus the miR-195 mimic group. &p < 0.05 versus the miR-195-inhibitor group. @p < 0.05 versus the pcDNA-LINC00461 + miR-195 mimic group.

To study the regulatory mechanism of LINC0046 on EMT, we constructed the LINC00461 overexpression plasmid (pcDNA-LINC00461) + miR-195 mimic, siLINC00461 + miR-195-inhibitor, pcDNA-LINC00461 + si-HOXA10 in FADU cells. Subsequently, we intended to identify the involvement of miR-195 and HOXA10 in the regulation of HNSCC by LINC00461. The transfection efficiency of LINC00461, miR-195, and HOXA10 was successfully validated by means of qRT-PCR (Figure 6A). Subsequent detection by immunofluorescence and western blot analysis (Figures 6B–6D) revealed an increased protein expression pattern of E-cadherin in cells transfected with miR-195 mimic or those co-transfected with miR-195 mimic and si-HOXA10, accompanied by the reduced protein expression patterns of Vimentin and Snail, compared with the blank control. In the presence of miR-195 mimic, the protein expression pattern of E-cadherin was elevated, and the expression patterns of Vimentin and Snail were downregulated by co-transfection with si-HOXA10, whereas the findings were annulled by subsequent treatment with pcDNA-LINC00461. The protein expression pattern of E-cadherin was reduced in cells transfected with siLINC00461 + miR-195-inhibitor or those treated with pcDNA-LINC00461 + si-HOXA10, accompanied by the increased protein expression patterns of Vimentin and Snail. The results obtained from the Transwell assay demonstrated suppressed migration and invasion abilities in those transfected with miR-195 mimic or those co-transfected with miR-195 mimic and si-HOXA10 compared with the blank control. In presence of miR-195 mimic, the migration and invasion abilities were suppressed due to co-transfection with si-HOXA10, which were neutralized by LINC00461 overexpression. The stimulated migration and invasion abilities were evident in the cells transfected with siLINC00461 + miR-195-inhibitor or those treated with pcDNA-LINC00461 + si-HOXA10 (Figures 6E and 6F). The aforementioned data suggested that LINC00461 elevated the expression pattern of HOXA10 by inhibiting miR-195 and subsequently stimulated the EMT of HNSCC cells.

### LINC00461 upregulates HOXA10 to enhance chemoresistance of HNSCC cells to cisplatin by inhibiting miR-195

**Figure 7:**
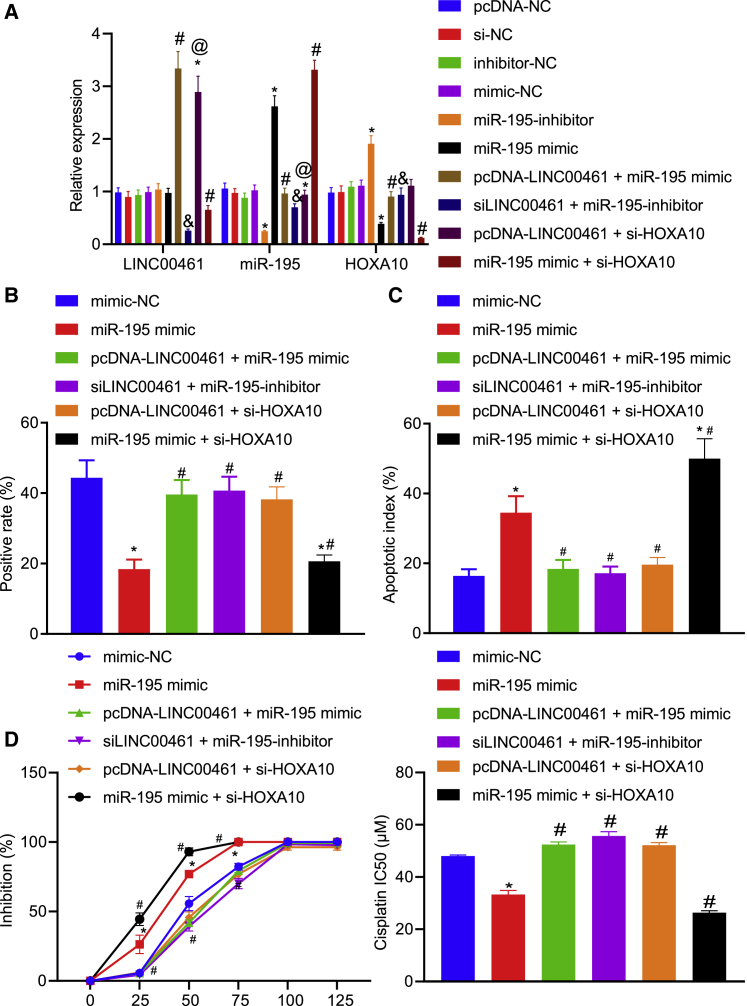
The LINC00461/miR-195/HOXA10 axis mediates chemoresistance of HNSCC cells (A) The expression of LINC00461, miR-195, and HOXA10 in the FADU cells detected by qRT-PCR. (B) The proliferative capacity of FADU/DDP cells. Red fluorescence represents proliferated cells and blue fluorescence represents nuclei. (C) The apoptosis rate of FADU/DDP cells. (D) Dose detection and data statistics of cisplatin IC_50_ following varied treatments. All of the data were measurement data expressed as means ± standard deviations. The data among multiple groups were compared using 1-way ANOVA. The experiment was conducted 3 times independently. ∗p < 0.05 versus the mimic-NC group. #p < 0.05 versus the miR-195 mimic group. &p < 0.05 versus the miR-195-inhibitor group. @p < 0.05 versus the pcDNA-LINC00461 + miR-195 mimic group.

To explore how LINC0046 enhanced the chemotherapy resistance of HNSCC cells to cisplatin based on the regulatory mechanism, we constructed pcDNA-LINC00461 + miR-195 mimic, siLINC00461 + miR-195-inhibitor, pcDNA-LINC00461 + si-HOXA10, or miR-195 mimic + si-HOXA10 in the FADU/DP cells. The transfection efficiency of LINC00461, miR-195, and HOXA10 was validated by qRT-PCR (Figure 7A). Through a combination of EdU (Figure 7B) and TUNEL assays (Figure 7C), the proliferation ability of the FADU/DDP cells transfected with miR-195 mimic or those transfected with miR-195 mimic + si-HOXA10 were evidently inhibited, while the apoptosis rate was increased. In comparison to the cells transfected with miR-195 mimic, the proliferation ability of the cells co-transfected with pcDNA-LINC00461 and miR-195 mimic, siLINC00461, and miR-195-inhibitor or pcDNA-LINC00461 and si-HOXA10 were enhanced, while the apoptosis rate was decreased, indicating that LINC00461 inhibited miR-195 to increase the chemoresistance of HNSCC to cisplatin. Moreover, compared to the cells transfected with miR-195 mimic, the cells co-transfected with miR-195 mimic and si-HOXA10 exhibited suppressed the proliferation ability and elevated the apoptosis rate. miR-195 targeted and downregulated HOXA10, thus reducing the chemoresistance of HNSCC to cisplatin. In addition, as depicted in Figure 7D, the chemoresistance of HNSCC cells to cisplatin was reduced in response to miR-195 mimic transfection compared to the mimic-NC transfection. Although the co-transfection with pcDNA-LINC00461 and miR-195 mimic enhanced the chemoresistance of HNSCC cells to cisplatin, it was annulled by dual transfection with miR-195 mimic and si-HOXA10. These results show that LINC00461 elevated the expression of HOXA10 by reducing the expression of miR-195, and then facilitated the chemoresistance of HNSCC cells to cisplatin.

### LINC00461/miR-195/HOXA10 affects the tumorigenicity of HNSCC cells *in vivo*

**Figure 8:**
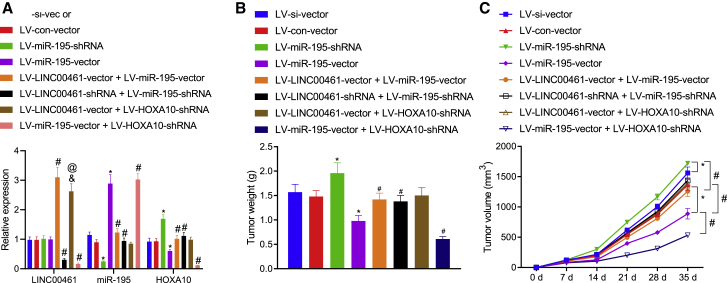
Tumorigenesis in mice is enhanced by LINC00461 by blocking miR-195-mediated inhibition of HOXA10 (A) The expression of LINC00461, miR-195, and HOXA10. (B) Tumor weight in nude mice. (C) Tumor volume in nude mice. All of the data were measurement data expressed as means ± standard deviations. The data among multiple groups were compared using 1-way ANOVA or repeated-measures ANOVA. n = 7 for BALB/c-nu mice following each treatment per experimental xenograft. ∗p < 0.05 versus the LV-si-vector or LV-con-vector group. #p < 0.05 versus the LV-miR-195-shRNA or LV-miR-195-vector group. &p < 0.05 versus LV-LINC00461-vector + LV-miR-195-vector group. @p < 0.05 versus the LV-LINC00461-vector + LV-miR-195-shRNA group.

To further explore the fundamental role of the LINC00461/miR-195/HOXA10 axis *in vivo*, xenograft tumor models were established and injected with the LV-LINC00461-vector + LV-miR-195-vector, LV-LINC00461-shRNA (short hairpin RNA) + LV-miR-195-shRNA, LV-LINC00461-vector +LV-HOXA10-shRNA group, or LV-miR-195-vector +LV-HOXA10-shRNA, respectively. qRT-PCR successfully validated the infection efficiency of LINC00461, miR-195, and HOXA10 (Figure 8A). Accordingly, our results from the subcutaneous tumor-bearing mice models demonstrated that the tumor weight and volume had decreased, with a slowed growth rate due to the lentivirus-mediated elevation of miR-195. Moreover, in the presence of miR-195, the lentivirus-mediated silencing of HOXA10 by shRNA further reduced the tumor weight and volume and slowed the overall growth rate. Moreover, the inhibited tumor growth and volume via the elevation of miR-195 was reversed by the overexpression of LINC00461. Increased tumor weight and volume and a stimulated growth rate were evident in response to LV-LINC00461-shRNA + LV-miR-195-shRNA or LV-LINC00461-vector + LV-HOXA10-shRNA (Figures 8B and 8C). The aforementioned findings indicated that LINC00461 accelerated the tumorigenicity of HNSCC cells *in vivo* via the elevation of HOXA10 by binding to miR-195.

## Discussion

Recently, the inhibition or reversion of EMT has been identified as an auspicious strategy for preventing cancer progression by comprehensively suppressing tumor cell invasion and cancer metastasis in certain malignancies, including HNSCC.^31^ The present study focused on unraveling the underlying mechanism of the LINC00461/miR-195/HOXA10 axis, contributing to the induction of EMT and the regulation of chemoresistance in HNSCC cells. Our findings revealed that the overexpression of LINC00461 could downregulate the miR-195 expression and subsequently upregulate the expression of HOXA10, thereby inducing the EMT and resistance of HNSCC cells to chemotherapy.

We initially reported that LINC00461 was highly expressed in the HNSCC cells and tissues, whereas the silencing of LINC00461 inhibited the EMT, migration, and invasion ability of HNSCC cells. Cancer cells typically manifest with EMT as a measure to advance the invasiveness and start metastasis.^32^ Recently, astudy highlighted the ability of LINC00461 knockdown to impair the EMT in non-small cell lung cancer cells.^33^ E-cadherin, Vimentin, and Snail are known as EMT markers.^34^ Notably, in the present study, a reversal of EMT due to LINC00461 knockdown has been identified by the upregulation of E-cadherin and the downregulation of Vimentin and Snail. Accumulating evidence supports the observation of a high expression of LINC00461 in several cancers; thus, silencing its expression could inhibit cancer progression. Accordingly, Yang et al.^10^ have revealed that the loss of LINC00461 prevents cell proliferation, migration, and invasion in glioma. Partially in consistency with our findings, Dong et al.^35^ found that LINC00461 was essentially upregulated in breast cancer (BC) tissues and cells, while its inhibition could reduce the expression of Vimentin but elevate the expression of E-cadherin. Moreover, a study by Ji et al.^36^ demonstrated that LINC00461 silencing resulted in inhibited tumor cell proliferation, migration, and invasion ability in hepatocellular carcinoma. In addition, the knockdown of LINC00461 strikingly impeded the proliferation and enhanced the apoptosis of multiple myeloma cells.^37^ Intriguingly, the essentiality of aberrantly expressed lncRNAs in cisplatin resistance has been highlighted in multiple cancers. Alternatively, siRNA-mediated LINC00461 silencing could decrease the resistance of renal cell carcinoma cells to sunitinib.^21^ The deficiency of LINC00461 was consequent for the increased sensitivity of rectal cancer cells to cisplatin and ultimately resulted in delayed rectal cancer progression.^38^

Our findings from RNA pull-down and RIP assays identified the ability of LINC00461 to bind to miR-195, while the dual-luciferase reporter assay confirmed that miR-195 targeted HOXA10. Consistently, it has been manifested that the binding of lncRNAs to miRNAs could promote the expression of mRNAs by blocking the degradation or translational inhibition of RNA induced by miRNAs.^39^ Moreover, existing studies have validated the capacity of LINC00461 to downregulate the expression of other anti-cancer miRNAs to upregulate their target genes, thereby facilitating cancer progression. For instance, Ji et al.^36^ reported that LINC00461 promotes the growth and invasion of hepatocellular carcinoma through the upregulation of leucine-rich and immunoglobulin-like domains 2 (LRIG2) by binding to miR-149-5p. Moreover, Zhou et al.^40^ have identified the cancer-promoting effect of lncRNA PVT1 initiated by binding to miR-195. Zuo et al.^41^ found that the binding of LINC00485 (a lncRNA) to miR-195 could radically reduce the chemosensitivity of lung adenocarcinoma cells to cisplatin by facilitating cell proliferation and repressing apoptosis. However, lncRNA CYTOR reduces radiosensitivity and induces the proliferative, migrating, and invading potentials of non-small cell lung cancer cells by binding to the miR-195.^42^

Essentially, miR-195 functions as an anti-neoplastic miRNA by regulating the migration and invasiveness of gastric cancer cells *in vitro*, while HOXA10 has been identified as a target of miR-195.^43^ Moreover, HOXA10 plays a cancer-promoting role in HNSCC cells. Accordingly, Guo et al.^20^ have exemplified that HOXA10, targeted by miR-135a-5p, is overexpressed in HNSCC cells, and the inhibition of its expression can inhibit tumor growth *in vivo* and cell proliferation *in vitro*. Furthermore, the silencing of LINC00461 can evidently enhance the radiosensitivity of lung adenocarcinoma cells via miR-195-mediated HOXA10 downregulation.^44^ Consistently, in the present study, we elicited a mechanism in HNSCC cells to exhibit the correlation between LINC00461, miR-195, and HOXA10 levels and the expression of EMT markers.

**Figure 9:**
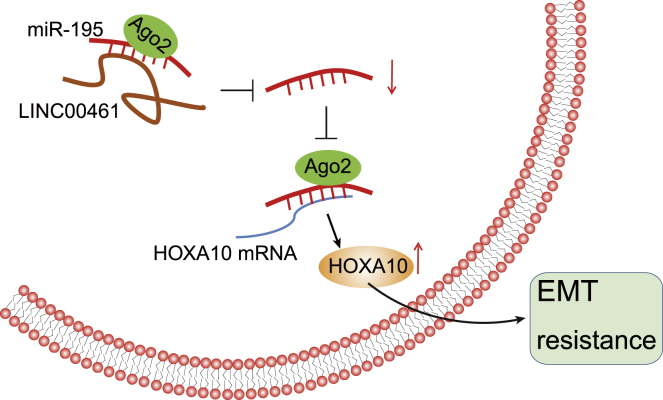
The graphical summary of the function and mechanism of LINC00461 in HNSCC LINC00461 competitively binds to miR-195 to upregulate HOXA10 expression, thereby inducing EMT and enhancing chemoresistance of HNSCC cells.

To conclude, this study verified that LINC00461 could enhance the progression of HNSCC and the chemoresistance to cisplatin by elevating the HOXA10 level via binding to miR-195 (Figure 9). With an elaborate understanding of their functions, LINC00461 can serve as a potential biomarker for the clinical treatment of HNSCC. However, those targets ascertain clinical validation for the potential development of RNA-based target therapies and to cope with chemoresistance.

## Materials and Methods

### Ethics Statements

This study protocol was performed with approval of the ethics committee of the Linyi People’s Hospital and in accordance with the Declaration of Helsinki. Written informed consent was provided by all of the participants or their guardians. All of the animal procedures were performed according to the *Guide for the Care and Use of Laboratory Animals* published by the US National Institutes of Health (NIH) with the approval of the research ethics committees (RECs) of the Linyi People’s Hospital. Adequate measures were taken to minimize the number and suffering of the included animals.

### Tissue samples and cell lines

The HNSCC and adjacent normal tissues were harvested from 52 patients (34 males and 18 females; aged 28–72 years, with a mean age of 50.63 ± 13.39) diagnosed with HNSCC at the Linyi People’s Hospital between March 2017 and March 2019. In accordance with the anatomical partition, clinical classification, and the classification standard of the Union for International Cancer Control (UICC), the patients were grouped as follows: 25 cases of laryngeal cancer (including 15 cases of supraglottic type, 10 cases of glottic type), 18 cases of hypopharyngeal carcinoma, and 9 cases of oropharyngeal cancer. Among them, 11 cases were in clinical stage I, 16 cases were in clinical stage II, 19 cases were in clinical stage III, and 6 cases were in clinical stage IV. Among these, a total of 30 cases of cervical lymph node metastasis and 22 cases without cervical lymph node metastasis were included. According to the 1998 World Health Organization (WHO) pathological grading criteria, the classification was as follows: 27 cases of well-differentiated SCC, 16 cases of moderately differentiated SCC, and 9 cases of poorly differentiated SCC. All of the HNSCC and adjacent tissues were incised into small pieces, quickly immersed in liquid nitrogen for storage, and transferred to a refrigerator at −80°C.

Throat SCC cell line Hep-2 (art. no. BNCC338610), tongue SCC cell line SCC-15 (art. no. BNCC340215), tongue SCC cell line Tca8113 (art. no. BNCC100956), nasopharyngeal SCC FADU (art. no. BNCC338343), and normal human esophageal squamous cell Het-1A (art. no. BNCC337688) were provided by the Beijing BeiNa Culture Collection (Beijing, China). Cells were cultured using Roswell Park Memorial Institute (RPMI) 1640 medium (GIBCO, USA) containing 10% fetal calf serum (GIBCO, USA) in an incubator (Thermo Scientific, USA) at a saturated humidity of 5% CO_2_ and 37°C.

### FISH assay

The localization of LINC00461 in HNSCC cells was identified by FISH using the Ribo lncRNA FISH Probe Mix (Red) (Ribo Bio, Guangzhou, China) in strict accordance with the provided instructions. Briefly, the cells were seeded in a 24-well culture plate at a density of 6 × 10^4^ cells per well and cultured until they attained 80% confluence. The cells were fixed using 1 mL 4% paraformaldehyde and then treated with proteinase K (2 μg/mL), glycine, and acetamidine reagent, followed by incubation with 250 μL pre-hybrid solution at 42°C for 1 h. Then, 250 μL hybridization solution containing the probe (300 ng/mL) was added to the cells for overnight incubation at 42°C. The cells were stained with DAPI (1:800) dye solution diluted with phosphate-buffered saline containing 0.1% Tween 20 (PBST) for 5 min. Finally, the cells were rinsed with PBST 3 times (3 min each time) and sealed with an anti-fluorescence quencher. Then, 5 different visual fields were randomly selected for observation and documentation under a fluorescence microscope (Olympus, Tokyo, Japan). The FISH Probe Mix for LINC00461 was synthesized by EXIQON (Vedbaek, Denmark), according to the specific sequence of 5′-GACATTTACGCCACAACCCACG-3′.

### Dual-luciferase reporter assay

To validate whether miR-195 specifically binds to LINC00461 and HOXA10, the artificially synthesized 3′ UTR fragment of HOXA10 and LINC00461 was introduced into the pMIR-reporter vector (Huayueyang Biotechnology, Beijing, China) using the endonuclease sites *SpeI* and *Hind III*. HOXA10 MUT and LINC00461 MUT plasmids were mutated on pMIR-reporter-HOXA10 and pMIR-reporter-LINC00461, respectively, and their individual MUT sequences were “AUUAAUAUUGUAAACGACCUG” and “AGTGCCTGGAGACAACTCCGCT.” The luciferase reporter plasmids WT and MUT were co-transfected into cells with miR-195. After 48 h of transfection, the cells were lysed, after which the luciferase activity was measured using a luciferase assay kit (K801-200, Biovision, Milpitas, CA, USA) on a Glomax 20/20 luminometer (Promega, Madison, WI, USA).

### RNA pull-down assay

Using a Magnetic RNA-Protein Pull-Down Kit (Pierce, Thermo Fisher, Austin, TX, USA), 1 μg biotin-labeled RNA was supplemented with 500 μL Structure Buffer followed by a 95°C water bath for 2 min. The RNA was then subjected to overnight incubation with 50 μL magnetic bead suspension at 4°C. The RNA-magnetic bead suspension was centrifuged at 3,000 rpm for 3 min with elimination of the supernatant. Then, the RNA-magnetic bead suspension was rinsed 3 times with 500 μL RIP Wash Buffer and incubated with 100 μL cell lysate for 1 h. The incubated magnetic bead-RNA-protein mixture was subsequently centrifuged at a low speed and eluted with 500 μL RIP Wash Buffer for 3 cycles. Finally, 10 μL of the cell lysate supernatant was used as input.

### RIP assay

The binding of LINC00461 to the Ago2 protein was detected using a RIP kit (Millipore, Danvers, MA, USA). Briefly, the cells were rinsed with pre-cooled PBS, lysed with the radioimmunoprecipitation assay (RIPA) lysis buffer (P0013B, Beyotime Biotechnology, Shanghai, China) in an ice bath for 5 min, and centrifuged at 14,000 rpm for 10 min at 4°C. A portion of the cell lysate was reserved as input, while the remaining portion was co-precipitated by incubation with antibodies against Ago2 (ab32381, 1:50, Abcam, Cambridge, UK), and immunoglobulin G (IgG) (1:100, ab109489, Abcam) as a NC. Comprehensively, 50 μL magnetic beads were resuspended in 100 μL RIP Wash Buffer and mixed with 5 μg of the corresponding antibody for 30 min. The magnetic bead-antibody complex was rinsed and resuspended in 900 μL RIP Wash Buffer, which was then incubated with 100 μL cell lysate overnight at 4°C. The magnetic bead-protein complex was eluted. The eluted sample and input were separately detached using proteinase K, after which the RNA content was extracted for subsequent PCR detection.

### Cell transfection

FADU and cisplatin-resistant FADU cells (FADU/DDP) (Hunan Fenghui Bio Technology, Hunan, China) were transfected with the pcDNA-NC plasmid, si-NC, mimic-NC, inhibitor-NC, pcDNA-LINC00461, si-LINC00461, miR-195 mimic, miR-195-inhibitor, and si-HOXA10 in combination or individually. Upon attaining 30%–50% cell confluence, the cells were transfected in a 6-well plate for 24–48 h according to the provided instructions of the Lipofectamine 2000 kit (Invitrogen, Carlsbad, CA, USA).

### qRT-PCR

The total RNA content was extracted from the tissues and cells using the TRIzol (Invitrogen), and its concentration and purity were then determined using a NanoDrop 2000 Spectrophotometer (1011U, NanoDrop, Wilmington, DE, USA). Then, the extracted RNA content was reverse transcribed into complementary DNA (cDNA) using the cDNA kit (K1622; Fermentas, Ontario, CA, USA) following the provided instructions. qRT-PCR was performed on an ABI 7500 instrument (Applied Biosystems, Foster City, CA, USA). The level of miR-195 relative to U6 and that of LINC00461 and genes relative to glyceraldehyde-3-phosphate dehydrogenase (GAPDH) were determined based on the 2^−ΔΔCt^ method: ΔΔCT = ΔCt_experimental group_ − ΔCt_Blank group or NC group_, wherein ΔCt = Ct_target gene_ − Ct_internal reference_. Primers were synthesized by TaKaRa Bio (Dalian, China) (Table 2).

**Table 2:** Primer sequences for qRT-PCR

Target	Sequence
LINC00461	F: 5′-GGAATCTTAAGCGCGGCAAG-3′
	R: 5′-AACAACTCGTTCCCACACA-3′
miR-195	F: 5′-GGGGAGCCAAAAGGGTCATCATCT-3′
	R: 5′-GAGGGGCCATCCACAGTCTTCT-3′
HOXA10	F: 5′-GCCCTTCCGAGAGCAGCAAAG-3′
	R: 5′-AGGTGGACGCTGCGGCTAATCTCTA-3′
U6	F: 5′-CGCTTCGGCAGCACATATACTA-3′
	R: 5′-CGCTTCACGAATTTGCGTGTCA-3′
GAPDH	F: 5′-TGGGTGTGAACCATGAGAAG-3′
	R: 5′-GCTAAGCAGTTGGTGGTGC-3′

F, forward; HOXA10, homeobox A10; LINC00461, long intergenic non-protein coding RNA 00461; miR-195, microRNA-195; qRT-PCR, quantitative reverse transcription-polymerase chain reaction; R, reverse; U6, small nuclear RNA.

### Western blot analysis

After 48 h of transfection, the cells were lysed by protein lysis buffer and centrifuged at 12,000 rpm and 4°C for a total of 20 min. Next, protein separation was conducted by electrophoresis and transferred onto nitrocellulose membranes. Membrane blockade was conducted using 5% skim milk and probed at 4°C overnight with the corresponding primary antibodies to rabbit anti-E-cadherin (1:500, ab15148), Vimentin (1:1,000, ab137321), and Snail (1:1,000, ab216347) from Abcam (Cambridge, UK). The membranes were re-probed with the horseradish peroxidase (HRP)-labeled goat anti-rabbit IgG (1:10,000, ab6728, Boster Biological Technology, Wuhan, China) for 1 h at 37°C. The membrane was rinsed with Tris-buffered saline with Tween (TBST) and developed with enhanced chemiluminescence (ECL). Gray-value analysis of the target bands was performed using the ImageJ (NIH, Bethesda, MD, USA) software.

### Immunofluorescence staining

After 48 h of transfection, the cells were fixed in 4% paraformaldehyde for 30 min. After treatment with 0.2% Triton X-100 for 15 min, the cells were blocked using 3% bovine serum albumin (BSA) at 4°C for 30 min and incubated with the primary antibody to E-cadherin (1:500, ab15148, Abcam), Vimentin (1:500, ab137321, Abcam) and Snail (1:250, MA5-14801, Invitrogen) overnight at 4°C. Fluorescein isothiocyanate (FITC)-labeled secondary goat anti-rabbit IgG (ab6717, 1:1,000, Abcam) was supplemented to the cells for another 2-h regimen of incubation at ambient temperatures under conditions devoid of light. Next, the cells were stained with DAPI (ab104139, 1:100, Abcam) for 10 min under conditions devoid of light. Finally, the cells were sealed and observed under an inverted fluorescence microscope (IX53, Olympus Optical, Tokyo, Japan).

### EdU assay

The cells were incubated with the EdU solution for 2 h, fixed using 40 g/L paraformaldehyde for 30 min, and incubated with glycine for 8 min. The cells were rinsed with PBS containing 0.5% Triton X-100 followed by staining with the Apollo staining solution for 30 min in conditions devoid of light. Finally, the cells were stained with Hoechst 3334 for 20 min. Under a fluorescence microscope, the red light of the excitation channel at a wavelength of 550 nm was used for photography, while the cells stained in red were considered the proliferative cells; the purple light of the excitation channel at a wavelength of 350 nm was used for photography, and the cells stained in blue were regarded as total cells. Cell proliferation rate = the number of proliferating cells (EdU^+^)/the number of total cells (Hoechst 33342^+^) × 100%.

### TUNEL assay

The paraffin slices were incubated with 50 μL TUNEL solution (Roche, Basel, Switzerland) for 50 min. The slices were incubated at 37°C for 30 min with 50 μL conversion agent POD 3 times. Next, 100 μL diaminobenzidine was added to the slices and developed for 10 min. The slices were subsequently counterstained using hematoxylin for 3 s. Then, the slices were sealed with neutral gum and observed under a microscope.

### Transwell assay

Upon attaining 80% cell confluence, the cells at passage 3 were starved in serum-free Dulbecco’s modified Eagle’s medium (DMEM) for 24 h. Serum-free DMEM was supplemented to the basolateral chamber of the Transwell chamber (Corning, Corning, NY, USA), under conditions of 37°C for 1 h. The cells were resuspended in serum-free DMEM. Then, 100 μL cell suspension (3 × 10^5^ cells/mL) was added to the apical Transwell chamber and 600 μL DMEM medium supplemented with 10% serum was added to the basolateral chamber. After 24 h of incubation, the cells were immersed in pre-cooled methanol for 30 min. Cells that had transferred to the basolateral chamber were stained with 0.1% Crystal Violet for 10 min. The migrated cells were observed and documented under an inverted microscope (Olympus).

The cell invasion experiment was conducted using the Matrigel-coated Transwell chamber. The cell concentration was adjusted to 1.0 × 10^5^/mL, and the other procedures were similar to the cell migration assay.

### Lentivirus construction and package

The miR-195 mimic, LINC00461 overexpression, and HOXA10- shRNA sequences were synthesized by Sangon Biotechnology (Shanghai, China) and introduced into the target vector pLVX-IRES-ZsGreenl or pLVX-shRNA, respectively. Lentiviral vector identification was conducted after vector transformation into DH5α. The lentivirus vector production was attained using the recombinant plasmid pLVX-miR-195-IRES-ZsGreenl (10 μg), pLVX-LINC00461-ZsGreenl (10 μg), or pLVX-shRNA-HOXA10 (10 μg) and the helper plasmids pspax2 (5 μg) and pMD2G (5 μg) (Invitrogen). High-purity endotoxin-free recombinant plasmids were extracted and then transduced into the 293T cells. After infection for 8 h, the medium was replaced with complete medium. Simultaneously, the empty plasmids were co-transduced into the 293T cells. After 48–72 h of transduction, the cell supernatants containing recombinant lentiviral particles were collected and concentrated to generate high-titer lentivirus. The lentiviruses with a titer >10^7^ TU/mL were stored in a refrigerator at −80°C for subsequent experimentation.

### Tumor formation in nude mice

Twenty-eight male BALB/c-nu nude mice (aged 3–5 weeks, weighing 16–20 g) were acquired from the Experimental Animal Center of Guangxi Medical University (Guangdong, China). The cells were infected with the lentiviruses expressing empty vector, miR-195, LINC00461, or HOXA10-shRNA, namely, LV-CON-vector, LV-miR-195-vector, LV-LINC00461-vector, and LV-HOXA10-shRNA, respectively, individually or in combination. The infected cells were subjected to green fluorescent labeling, and the labeled cells in the logarithmic growth phase were trypsinized and counted. A total of 0.2 mL cell suspension (1.0 × 10^7^ cells/mL) was subcutaneously injected into the nude mice at the right hind limb groin. The growth of xenografts was monitored, with the tumor diameter measurement once per week for 5 consecutive weeks. On the 35^th^ day, the number of formed tumors in the nude mice was documented. The mice were then euthanized and the excised tumors were weighed and photographed.^45^

### Statistical analysis

All of the data were processed using the SPSS version 21.0 statistical software (IBM SPSS Statistics, Armonk, NY, USA). The measurement data were expressed as means ± standard deviations. The comparison between HNSCC and the adjacent normal tissues was performed using the paired t test, while comparison between the other two groups was conducted by the unpaired t test. Data among multiple groups were compared using a one-way analysis of variance (ANOVA) with Tukey’s post hoc tests. The tumor volume at different time points was analyzed using repeated-measures ANOVA. A value of p <0.05 was indicative of a statistically significant difference.

## Supplemental information

Document S1. Figure S1

Document S2. Article plus supplemental information
