# Supplementary material for: LINC00461 facilitates HNSCC development and reduces chemosensitivity by impairing miR-195-mediated inhibition of HOXA10
Source: Mol Ther Oncolytics. 2021 Jan 20;21:74–86. doi: 10.1016/j.omto.2021.01.008 (PMC8027536; doi:10.1016/j.omto.2021.01.008)
Supplement: Document S1. Figure S1 [file mmc1.pdf]

OMTO, Volume *21*

Supplemental information

LINC00461 facilitates HNSCC development and reduces chemosensitivity by impairing miR-195-mediated inhibition of HOXA10

Yifang Guan, Aizhong Guan, Long Chen, and Aimei Gong

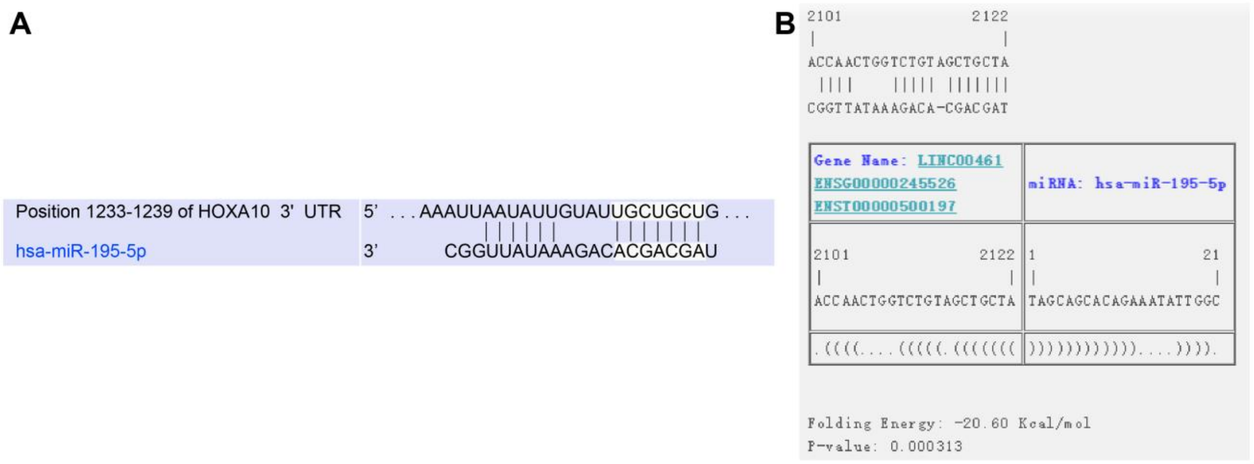

Supplementary Figure 1. The binding relationship between LINC00461, miR-195, and HOXA10. A, Putative miR-195 binding sites in the 3'UTR of HOXA10 mRNA in the TargetScan database (http://www.targetscan.org/vert_71/). B, Putative miR-195 binding sites in LINC00461 in the RNA22 database (https://cm.jefferson.edu/rna22/).
